# Heavy metal contamination in sediments of the Sematan-Serayan River, Sarawak: Assessment of pollution indices and environmental risk implications

**DOI:** 10.1007/s10661-026-15110-w

**Published:** 2026-02-24

**Authors:** Farah Akmal Idrus, Valerie Urai Ding

**Affiliations:** Faculty of Resource Science and Technology, Universiti Malaysia Sarawak (UNIMAS), 94000 Kota Samarahan, Sarawak, Malaysia

**Keywords:** Heavy metals, Sediment pollution indices, Sematan-Serayan River Sarawak, Total organic carbon, Anthropogenic sources

## Abstract

Sediments in river systems act as both sinks and sources of pollutants, which can have significant environmental and health implications. This study aimed to assess the concentrations of heavy metals in relation to total organic carbon (TOC) in the sediments of the Sematan-Serayan River, Sarawak, Malaysia. Sediment samples were collected in November 2024. Heavy metal and TOC concentrations were analysed via flame atomic absorption spectroscopy and a TOC analyser, respectively. The average heavy metal concentrations decreased in the order of Fe > Al > As > Zn > Ni > Cr > Pb > Mn > Cu > Co > Cd. Higher heavy metal concentrations associated with TOC were observed, particularly at stations close to anthropogenic activities, indicating pollution hotspots. The surface sediments were dominated by sand (> 90%). Pollution indices, including the contamination factor (CF), geoaccumulation index (Igeo), pollution load index (PLI), and potential ecological risk index (RI), were used to assess the severity of environmental pollution. All the stations were highly contaminated (CF > 6) and moderately to strongly polluted by Pb and As (Igeo > 2). Moreover, Stations 1 and 3–7 were considered deteriorated zones during the monsoon season (PLI > 1). The RI was very high (RI > 600) for Pb, As, Cu, Zn, and Cr at all stations. A comparison of the pollution indices with hierarchical cluster analysis suggested that anthropogenic activities were the main sources of heavy metal contamination in this river, potentially disturbing the biological functions of the benthos.

## Introduction

Rivers are among the most important aquatic environments that may sustain a wide variety of life forms, including fish, invertebrates, microbes, terrestrial and semiaquatic animals. Rivers also serve as a significant biogeochemical cycle that supports global carbon cycles (Battin et al., 2023), particularly in mangrove areas. River runoff may carry loose soils from the land, which then slowly sink to riverbeds as sediments (Zarfl & Dunn, 2022). Sediments can shape a river's structure, which can have an impact on the environment and the flow of pollutants and nutrients to the sea. Sediments are essential for carbon sequestration because they are naturally occurring carbon sinks (Liu et al., 2023a, b). Carbon in surface sediment indicates the abundance of organic matter, which provides microbial populations with energy to continue the nutrient cycle process (Li et al., 2024; Wang et al., 2023). Carbon is usually stored at different depths in sediment. The top layer of sediment (0–10 cm) contains labile organic carbon that decomposes easily, whereas organic carbon becomes more stable at 10–15 cm, which helps in long-term sequestration (Zhang et al., 2021).

Heavy metals can exist in river sediments due to anthropogenic activities, such as industrial, agricultural, and urbanisation processes (Liao et al., 2024). Certain heavy metals, such as Fe, Mn, and Co, which are required for physiological processes, are essential to biota at trace concentrations. Though, some other heavy metals, such as Pb, As, and Cd, are considered toxic even at small concentrations. The relationships between total organic carbon (TOC) and heavy metals in estuary sediments can differ according to location and source. When the correlation between TOC and heavy metals is weak, it can indicate that the heavy metals are derived from terrestrial sources. Moreover, a strong correlation between TOC and heavy metals suggests that organic matter may increase metal accumulation in sediments (Sevastyanov et al., 2020). The influence of seawater intrusion or coastal inputs can further contribute to elevated contamination levels due to the transport and deposition of metal-laden particulates and dissolved substances from oceanic currents and tidal actions (Zhang et al., 2024).

Sand extraction from riverbeds is needed to meet the high demand for use in construction and industry, such as buildings, glassmaking, and land reclamation. River sand dredging/mining activities in Sarawak are permitted by the Sarawak Land and Survey Department but must follow strict guidelines set by the Department of Irrigation and Drainage Sarawak (DID, 2025). However, extensive and excessive riverbed removal activities have caused extensive environmental destruction, such as in the Mekong Delta, Vietnam (Park, 2024); the Odor River, Nigeria (Akanwa, 2020); and the Pussur River, Bangladesh (Rahman and Ali, 2024). River sand dredging causes riverbank instability (Hackney et al., 2020), endangering bridges and increasing flooding in nearby areas, thus increasing contaminant flushing into the water body. It also increases saltwater intrusion in deltaic and coastal areas (Park et al., 2021), which can harm crops such as paddy rice and reduce river biodiversity.

This study aimed to assess the concentrations of heavy metals and TOC in the surface sediment of the Sematan-Serayan River, Sarawak, Malaysia, with a focus on the factors that influence their sources by investigating pollution indices and correlation studies to provide an understanding of the implications of heavy metals on environmental risk.

## Materials and methods

### Study sites and sample collection

**Fig. 1:**
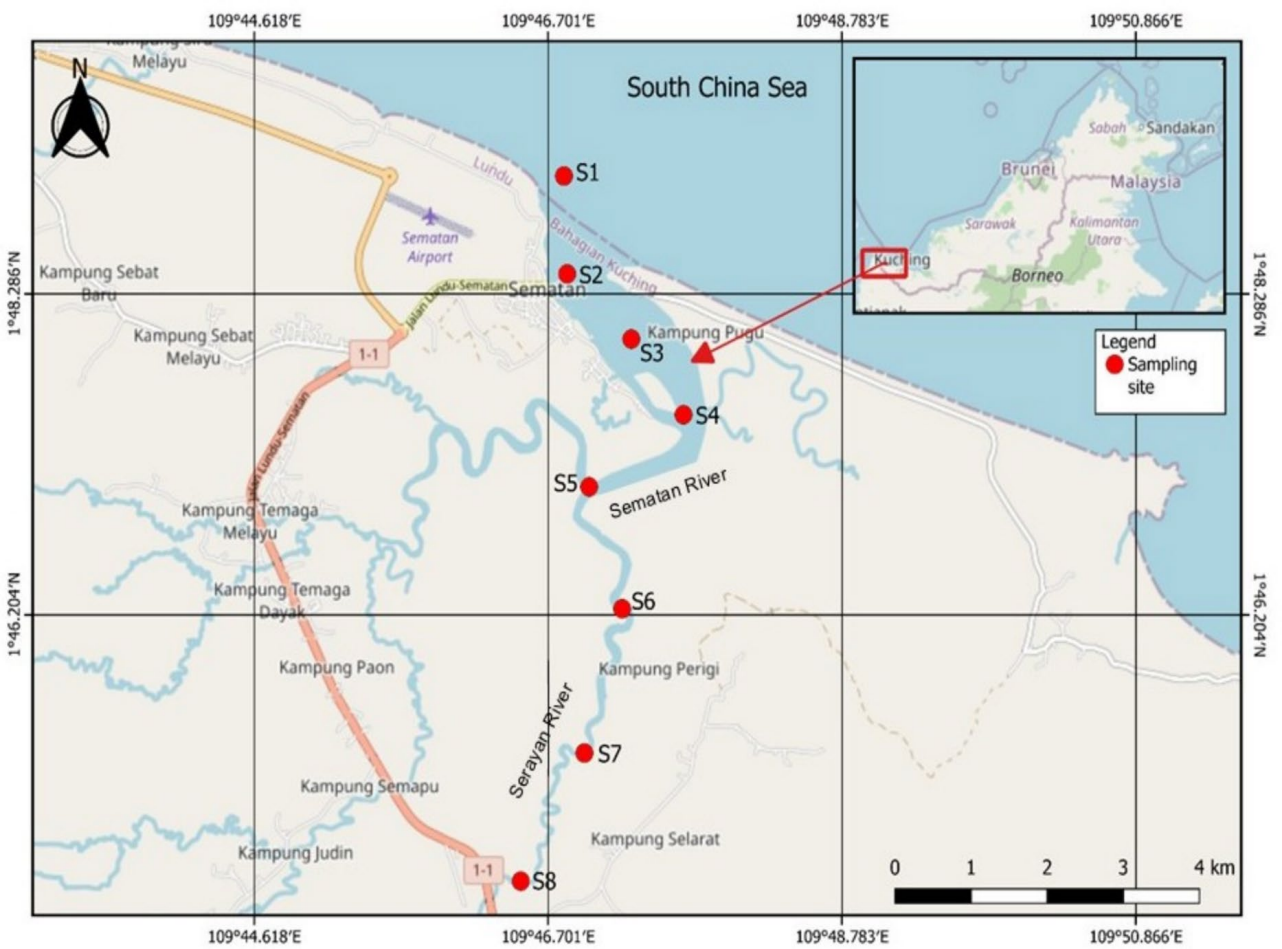
The sampling stations at the Sematan-Serayan River are plotted with red dots. S1–S5 are located at the Sematan River, while S6–S8 are located at the tributary of the Sematan River (Serayan River). A map of the study site was created via the Quantum Geographic Information System (QGIS 3.40.0)

Sample collection was carried out on the 9th of November 2024, during the wet (monsoon) season, along the Sematan-Serayan River, Sarawak, Malaysia (Fig. 1), from 1°48.7487' N, 109°46.7984' E to 1°44.4650' N, 109°46.4421' E. The Sematan-Serayan River is located between the Lundu and Sematan regions, near the western end of Sarawak, and flows directly into the South China Sea. There are several human activities along this river, such as sand dredging, agricultural activities, palm oil plantations, and chalet activities (in coastal areas). In addition, there are mangrove and blackwater areas. Sediment samples for heavy metal, TOC, and particle size analyses were collected from eight sampling stations, where Stations 1–5 (S1–S5) were located at the Sematan River, and Stations 6–8 (S6–S8) were located at the tributary of the Sematan River, which is the Serayan River. Surface sediment samples were collected from the top 0–5 cm of the riverbed via the Ekman grab. The sediment samples were placed in polyethylene zipper bags with labels and then kept in plastic containers with lids to avoid contamination during sampling. The plastic containers were stored in a cooler box with ice during transportation to the Aquatic Chemistry Laboratory at Universiti Malaysia Sarawak (UNIMAS). In the laboratory, the sediment samples were stored at −20 °C in a freezer prior to further analysis.

### Sample digestion and heavy metal analysis

The sediment samples were thawed before drying at 65–70 °C in an oven for 24 h. Approximately 0.2 g of dried sediment was digested in a mixed concentrated acid mixture (nitric acid‑hydrogen peroxide-hydrochloric acid) following the standard method of the USEPA (2007) using a microwave digestion system (Anton Paar Multiwave GO). The heavy metal concentrations (Cu, Fe, Pb, Zn, Ni, Co, Cr, Cd, Mn, Al, and As) were analysed via flame atomic absorption spectroscopy (Analytik Jena novAA 800) in the Analytical Absorption Laboratory, Faculty of Resource Science and Technology, UNIMAS. All the readings were determined in triplicate. Certified reference materials (CRMs) from the National Research Council of Canada of MASS-4 were analysed using the same methods as those used for the sediment samples. The measured CRMs showed good agreement (90–110%) with the certified values, confirming the reliability of the data from this study. All chemicals used in this study were of analytical grade and were obtained from Merck or Fisher Scientific. Other glassware and plastic apparatuses were acid-washed prior to use. Nitrile nonlatex gloves or polyethylene gloves were used during sampling, handling, and laboratory work to avoid direct contact with the samples.

### Particle size analysis

The dry sieving method was employed following the procedure described by Holmes and MacIntyre (1984). The dried sediment samples were transferred to a stacked series of graded sand sieves, starting with mesh sizes of 1.0, 0.50, 0.25, 0.125, and 0.063 mm. The stacked column of sieves was placed in an automatic sieve shaker for 10–15 min. The sand fraction on each sieve was transferred to a preweighed dish, and the weight was measured using an electronic balance. The sediments were classified as silt–clay (≤ 0.063 mm), very fine sand (0.063–0.125 mm), fine sand (0.125–0.25 mm), medium sand (0.25–0.5 mm), coarse sand (0.5–1.0 mm), or very coarse sand (≥ 1.0 mm).

### Total organic carbon

The TOC content was determined using a TOC analyser (Shimadzu TOC-L) at the Analytical Chemistry Laboratory, Faculty of Engineering, UNIMAS. Each sample was measured in triplicate. Prior to conducting TOC analysis, the samples were digested following the standard method EPA 3051A (USEPA, 2007).

### Assessment of ecological risks

#### Geoaccumulation index

The geoaccumulation index (Igeo) was measured to determine the accumulation level of metals in sediments from natural and anthropogenic sources by comparing the metal concentrations in this study with the background values using Eq. 1:

1 $$Igeo = {\mathrm{log}}_{2}\left(\frac{{\mathrm{C}}_{\mathrm{n}}}{1.5{\mathrm{B}}_{\mathrm{n}}}\right)$$

where C_n_ is the (individual) metal concentration (mg/kg) in this study, and B_n_ is the reference background value from soil adopted from Muller (1969) and Taylor (1964). A factor of 1.5 was introduced to reduce the effect of potential differences in the background values, possibly due to lithological variations in the sediment. The contamination classification values from Igeo are listed in Table 1.

**Table 1:** Classification of environmental assessment indices for heavy metal contamination in sediment

Igeo	CF	PLI	RI
< 0 Unpolluted, Class 0	< 1 Low contamination	< 1 Unpolluted	< 150 Low risk
0 ≤ Igeo < 1 Unpolluted to moderately polluted, Class 1	1 ≤ CF < 3 Moderate contamination	> 1 Polluted	150 ≤ RI < 300 Moderate risk
1 ≤ Igeo < 2 Moderately polluted, Class 2	3 ≤ CF < 6 Considerable contamination		300 ≤ RI < 600 Considerable risk
2 ≤ Igeo < 3 Moderately to strongly polluted, Class 3	> 6 Very high contamination		> 600 Very high risk
3 ≤ Igeo < 4 Strongly polluted, Class 4			
4 ≤ Igeo < 5 Strongly to extremely polluted, Class 5			
> 5 Extremely polluted, Class 6			

#### Contamination factor

Contamination factor (CF) was used to evaluate the level of pollution by indicating the degree of contamination (Ouattara et al., 2022). The CF was calculated using the formula of Muller (1969), which is shown in Eq. 2:

2 $$\mathrm{CF}=\frac{ {M}_{x}}{{M}_{b}}$$

where M_x_ is the concentration of a metal (individual) in the sample and M_b_ is the concentration from the reference background value (Taylor, 1964). The contamination classifications of the CF samples are listed in Table 1.

#### Pollution load index

Pollution load index (PLI) was used to assess the overall metal pollution status in a given area, taking into account multiple metals, on the basis of the formula by Tomlinson et al. (1980), by applying the CF values (Eq. 3):

3 $$\mathrm{PLI}={\left({\mathrm{CF}}_{1} \times {\mathrm{CF}}_{2} \times {\mathrm{CF}}_{2}\dots \dots . {\mathrm{CF}}_{\mathrm{n}}\right)}^{\frac{1}{\mathrm{n}}}$$

where CF_n_ is the contamination factor of the metal and n is the number of metals. The PLI classifications are listed in Table 1.

##### Potential ecological risk index

The potential ecological risk index (RI) was used to assess the environmental effects of contaminants accumulated in sediments (Hakanson, 1980a, 1980b). The RI was calculated using Eq. 4:

4 $$\mathrm{RI}= \sum\nolimits_{i=1}^{n}{\mathrm{E}}_{\mathrm{r}};\,where \,{(\mathrm{E}}_{\mathrm{r}}= {\mathrm{T}}_{\mathrm{r}} \times \text{ CF})$$

where CF is the contamination factor, E_r_ is the potential risk of an individual metal, and T_r_ is the toxic response of an individual metal. In Hakanson's approach (Hakanson, 1980a, b), the T_r_ values for As, Cd, Cr, Cu, Pb, and Zn are 10, 30, 2, 5, 5, and 1, respectively. The RI classifications are listed in Table 1.

### Statistical analysis

The data were analysed via IBM SPSS Statistics v.31.0 for the mean, standard deviation, and one-way analysis of variance (ANOVA) with Levene's test for homogeneity of variance to assess the significant differences in heavy metal concentrations and TOCs at all the stations, as well as to obtain descriptive statistics of particle sizes at all the stations. Pearson's correlation analysis and hierarchical cluster analysis (HCA) were also performed via IBM SPSS Statistics v.31.0. HCA was conducted on the datasets by applying Ward's method with Euclidean distances of similarity clusters for heavy metals and sampling stations.

## Results and discussion

### Concentration of heavy metals in the surface sediments of the Sematan-Serayan River

**Fig. 2:**
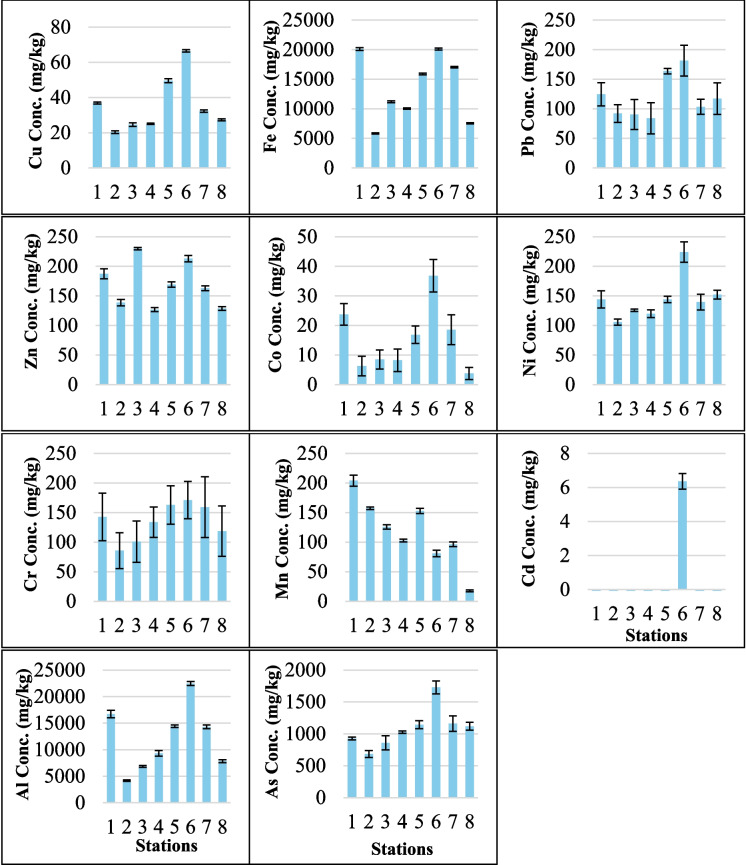
Concentrations of Cu, Fe, Pb, Zn, Co, Ni, Cr, Mn, Cd, Al, and As in surface sediments collected along the Sematan-Serayan River, with different scales used for metal concentrations. The concentration units are in mg/kg and the error bars represent the standard deviation

The average concentration of each metal followed the order of Fe > Al > As > Zn > Ni > Cr > Pb > Mn > Cu > Co > Cd. Strong positive correlations were observed between various heavy metals and associated elements in the surface sediments (p < 0.05), including Cu-Pb, Cu-Co, Cu-Ni, Cu-Al, Cu-As, Fe-Co, Fe-Al, Pb-Ni, Pb-As, Co-Al, Ni-As, and Cr-Al (Table 2). The concentrations of heavy metals in the surface sediments from the eight stations are plotted in Fig. 2 and ranged from 20.32 to 66.50 mg/kg (Cu), 5,838.00 to 20,093.01 mg/kg (Fe), 84.02 to 181.26 mg/kg (Pb), 126.68 to 229.87 mg/kg (Zn), 3.76 to 36.79 mg/kg (Co), 105.59 to 224.07 mg/kg (Ni), 85.56 to 171.01 mg/kg (Cr), 17.72 to 203.87 mg/kg (Mn), < 6.36 mg/kg (Cd), 4,168.65 to 22,467.49 mg/kg (Al), and 684.48 to 1,727.46 mg/kg (As).

**Table 2:**
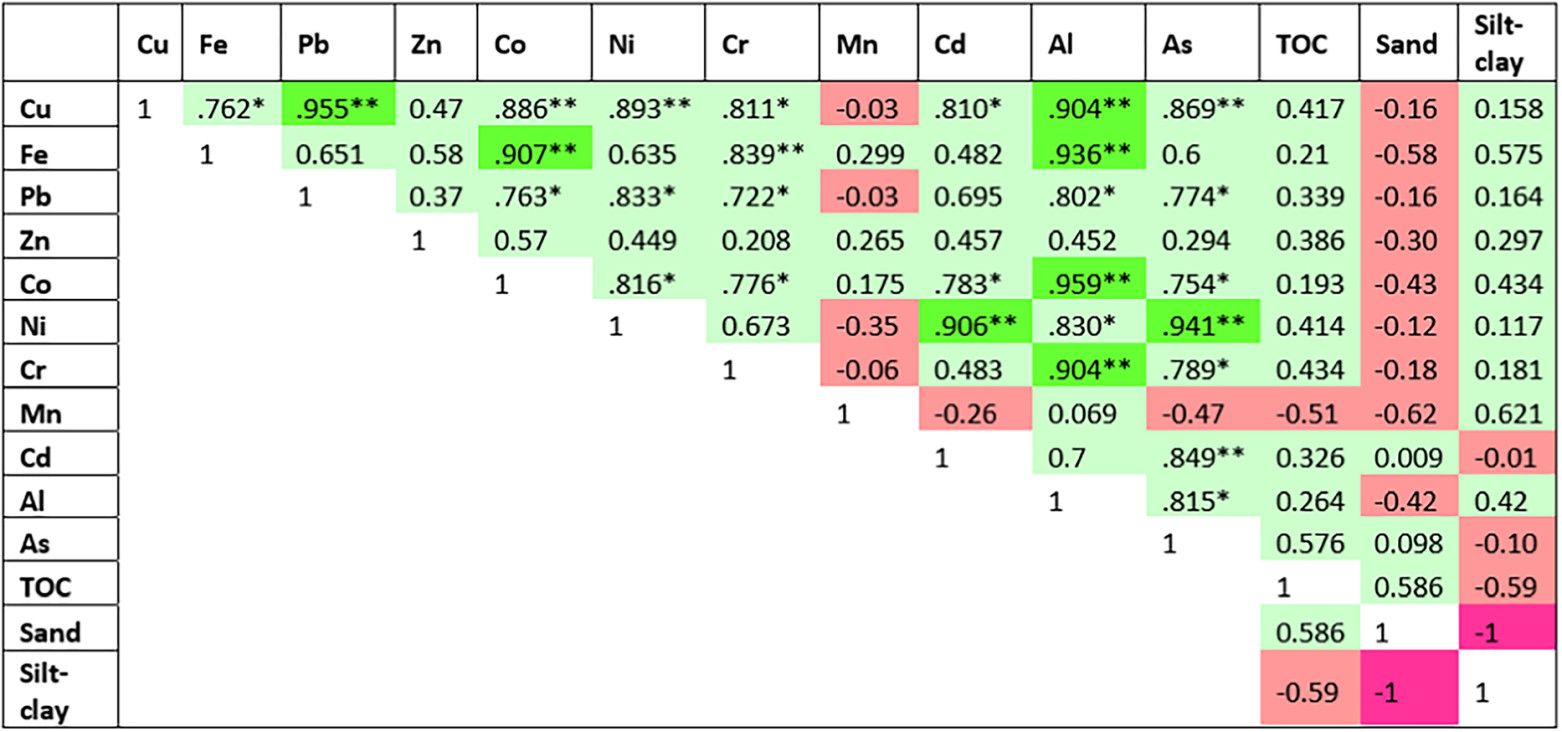
Pearson's correlation matrix of metals, TOC, sand (%), and silt–clay (%) in surface sediments from the Sematan-Serayan River (  Highly positive correlation,  positive correlation,  negative correlation, and  highly negative correlation)

^*. The correlation is significant at the 0.05 level (two−tailed).^

^**. The correlation is significant at the 0.01 level (two−tailed).^

The average concentration of Fe was the highest among all the metals, with S6 having the highest value (20,093.01 ± 175.29 mg/kg). The total Fe contents from S6 and S7 were released from sand dredging activities and bound with high organic carbon from the organic-rich waters at these stations (Wilkes et al., 2018). The high TOC content (Fig. 4) obtained in this study stimulates reductive dissolution, a process that can remobilise Fe into the sediment phase (Basti et al., 2025). In comparison, S2, which was near the river mouth, had a lower Fe concentration (5,837.99 ± 97.86 mg/kg), likely due to the influence of the salinity gradient (Fang & Wang, 2022; Zhang et al., 2023a, b), which led to the accumulation of metals in the sediments (José et al., 2021). At the river mouth station (S1), this active process also led to elevated concentrations of Fe, as particles settled and accumulated in this transitional zone. The oxidation state of Fe can vary greatly in estuarine environments, influencing its solubility and movement. For example, ferrous iron (Fe(II)) is more soluble and more readily transported than ferric iron (Fe(III)). The changes in Fe levels were influenced mainly by physical mixing and biogeochemical reactions during tidal fluctuations. In brackish zones, the main cause of the increase in Fe is the transformation of amorphous Fe(OH)_3_ into FeS. The cycling of Fe (hydr-)oxides is closely linked to that of organic carbon, which significantly affects the rate and pattern of organic carbon turnover in these ecosystems (Yu et al., 2021).

Mn experiences sediment resuspension and mixing between seawater and freshwater because seawater intrusion facilitates Mn partitioning into the particulate phases within the water column (Zhang et al., 2023a, b). However, the geochemical behaviour of Mn differs from that of Fe. In estuaries, Mn reduction occurs more readily and can even play a dominant role in the remineralisation of organic matter. Additionally, the formation of Mn(III)-humus complexes is notable in humus-rich estuaries, such as the Sematan-Serayan River, potentially influencing Mn cycling within the sediment (Cai et al., 2021). Therefore, the concentrations of Mn in sediment are usually low in low-salinity areas and increase with increasing salinity, as shown in this study (Fig. 2). Mn is a redox-sensitive element; hence, its mobility and concentration can vary significantly depending on the oxygen level (Berger et al., 2022). In anoxic sediment zones, especially near river mouths where organic matter decomposition depletes oxygen, Mn primarily exists in its reduced and soluble form, Mn(II), increasing its mobility within porewater (Ma et al., 2023). Under these low-oxygen conditions, insoluble manganese oxides (Mn(IV)) are chemically reduced and dissolve into interstitial water (Yang et al., 2022). As this soluble Mn(II) diffuses or is released into the overlying water, it contributes to increased concentrations near the sediment–water interface.

Al is a reliable tracer of atmospheric dust input in aquatic ecosystems, particularly in areas affected by industrial or agricultural activities. Airborne dust can be transported over long distances and deposited in distant watersheds, including aquatic environments (Idrus et al., 2021). The Al concentrations were high at all stations, with the highest concentration recorded at S6 (22,467.49 ± 381.20 mg/kg), which supports the idea of sediment entrapment and mineral input from extracted sediments from the sand dredging activity that leached into the river. Furthermore, this river (Sematan-Serayan River) is also near Munggu Belian and Gebong Hills in Sematan, which are ex-bauxite mining areas (Idrus et al., 2024; Lee et al., 2017). Bauxite is a rock such as pyroxene andesite, gabbro, and ferruginous greenstone, which consists of Al minerals with Al- and Fe-rich soils (Lee et al., 2017). Al enrichment in sediments may stem from erosion of lateritic soils, especially during rainfall events (Sarkar et al., 2020). Al can also be derived from lithogenic input because its high concentration may reflect natural soil material settling in low-energy areas such as those in S6. In contrast, S2 had much lower Al levels, which may reflect less sediment retention and higher flushing rates due to its location near the river mouth (Richard et al., 2020), with very strong prevailing winds and waves during the wet monsoonal season during sampling.

The highest As concentration was recorded at S6 (1,727.46 ± 102.90 mg/kg). Moderate positive correlations were observed for As-Fe and As-TOC (Table 2), but these correlations were not statistically significant (p > 0.05). These findings indicate that elevated As concentrations are associated with the presence of Fe oxides and organic matter in the sediment. As often adsorbs onto Fe oxides (Aftabtalab et al., 2022). High TOC (Fig. 4) can create reducing conditions that mobilise As by promoting the dissolution of Fe oxides and releasing bound As into the surrounding sediment (Miller et al., 2022; Yan et al., 2023). This is consistent with findings from other regions, such as the Buladu River in Indonesia, where As pollution has been linked to upstream gold mining (Basir et al., 2022), and the Ohinemuri River in New Zealand, where historic mining caused dangerously high As levels in sediments and streambanks (Clement et al., 2017). The Meghna River in Bangladesh also has high sediment As concentrations (> 500 mg/kg) in zones with strong sediment–water mixing (Kwak et al., 2024). In comparison, sediment from the Trująca River, Poland, reached 3,000 mg/kg near an abandoned gold-arsenic mine (Stachnik et al., 2020). Similarly, a study in coastal China revealed that human activities such as farming and waste disposal contributed to As pollution, although the average As concentration was much lower (9.75 mg/kg) (Liu et al., 2022a, b) (Fig. 2).

High Ni (224.07 ± 17.18 mg/kg), Cu (66.50 ± 0.65 mg/kg), and Pb (181.26 ± 26.09 mg/kg) concentrations suggest inputs from upstream agricultural sources, possibly fertilisers (Jahidin et al., 2020). Cu is known to bind with sulphides and organic matter (Hoffmann et al., 2020), both of which are expected to occur in the silt–clay sediment of S6 (Fig. 5) under anoxic conditions. The strong correlations of Cu-Pb (r = 0.956, p < 0.05) and Cu-Al (r = 0.904, p < 0.05) further suggest that terrestrial sources are linked to sediment input or historical deposition. Notably, site S6 was the only site where Cd was detected (0.46 mg/kg), indicating either episodic contamination or localised retention in fine sediments (El-Saadani et al., 2022).

The Cd concentrations are generally below the detection limit at all the stations except S6. This suggests low natural abundance, high solubility, and detection limitations in the instrumentation used. Cd tends to remain dissolved in water or bind loosely to sediments (El-Saadani et al., 2022). The presence of Cd at S6 likely reflects occasional inputs from upstream or past pollution events but may also be influenced by sand dredging activities in the area, which can resuspend contaminated sediments and release bound metals (Paula et al., 2024).

### Identification of heavy metal sources in sediments

**Fig. 3:**
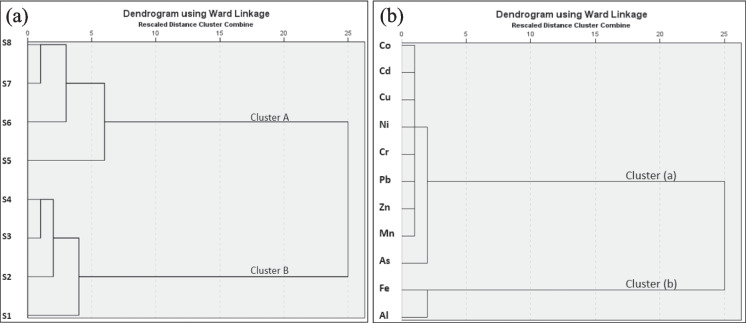
Dendrogram of sampling stations (**a**) and heavy metals (**b**) based on their total metal contents in surface sediments from the Sematan-Serayan River

Several natural and anthropogenic factors control the sources of heavy metals. The sources of total metal contents in surface sediments from the Sematan-Serayan River were examined via HCA (Fig. 3). Two distinct metal clusters were identified: (a) Co-Cd-Cu-Ni–Cr-Pb‒Zn-Mn-As and (b) Fe-Al. The metals in Cluster (b) were significantly correlated (r = 0.936, p < 0.01), indicating that their similar common sources were comparable with the background values, which were derived mainly from natural geological sources, and their distribution in sediments was dependent on hydrodynamic conditions. Moreover, the Sematan region has been identified as an ex-bauxite mining area (Lee et al., 2017), which may have influenced the Fe and Al levels found in sediments.

The comparison between stations revealed two clusters: Cluster A and Cluster B. Cluster A encompassed several stations (S5, S6, S7, and S8), primarily from areas near sand dredging, agricultural farms, and oil palm plantations, which received runoff inputs from fertiliser leaching. These high levels likely resulted from a combination of dredging-induced sediment resuspension and runoff from nearby plantations. Comparable dredging activity was reported in Lake Victoria, Africa, where sediments near sand extraction sites contained much higher metal concentrations (Baguma et al., 2022). The higher values at S5 and S6 for Cr, Pb, Cu, and Ni suggest more intense or poorly mitigated anthropogenic impacts in the study area. The notably high Cr levels support earlier findings that Cr strongly binds to fine sediments and organic matter in low-flow environments (Dhanakumar & Mohanraj, 2018). These observations also align with studies highlighting mangrove sediments as effective sinks for heavy metals because of their fine-grained nature (Fig. 5) and anoxic conditions (Mohammed et al., 2024). The sheltered, low-energy environment with dark brown and black, organic-rich waters at S7 and S8 likely contributed to metal retention through complexation with organic matter and fine sediments (Maldini et al., 2023). Blackwater, which has a low pH, high humic acid content, and slow sedimentation, may limit the accumulation of certain metals at S7 and S8. Both stations, especially S7, presented high concentrations of Al, Mn, and Fe, which is consistent with blackwater systems that promote metal leaching from riverbanks (Flotemersch et al., 2024; Li et al., 2024).

Cluster B, especially at the river mouth stations (S1 and S2), presented elevated concentrations of As, Pb, and Fe, which were influenced by a combination of tidal mixing, fluvial inputs, and sediment retention during slack water periods in estuarine sediments (Li et al., 2023; Liu et al., 2022a, b). Compared with those at the Tigris-Euphrates River mouth, where the Fe and Pb concentrations were reported to be 811.71 and 67.34 mg/kg, respectively (Al-Shawi et al., 2022), the Fe concentration at S1 was much greater. This suggests a stronger input of Fe-rich particulates, possibly from natural geological sources, as well as anthropogenic contributions such as iron debris from boats, industrial runoff, or other human activities along the river (Li et al., 2023).

Heavy metals in Cluster (a) were particularly high, especially in the Cluster A regions. The contents of Cluster (a) (Co, Cd, Cu, Ni, Cr, Pb, Zn, Mn, and As) were higher than their background values, reflecting contributions from anthropogenic inputs (i.e., sand dredging/mining). Sand mining is a known source of As contamination due to the disturbance caused by buried sediments, which can release both naturally occurring and human-influenced As into the environment (Patel et al., 2023). Strong positive correlations were observed between various heavy metals and associated elements in the surface sediments (p < 0.05), including Cu-Pb, Cu-Co, Cu-Ni, Cu-As, Fe-Co, Pb-Ni, Pb-As, and Ni-As (Table 2), suggesting a shared origin or common geochemical behaviour among these metals in the sediment matrix, potentially linked to the natural weathering of soil minerals (Fang et al., 2021). Cluster B contained S1, S2, S3, and S4, which were close to the river mouth, small jetties, and villages. No sand dredging or farming activities were detected in these regions. Thus, the concentrations of heavy metals in these regions were most likely from natural sources, such as rock weathering, precipitation, runoff from nearby lands, and river flow to the sea.

### Distributions of TOC in the surface sediments of the Sematan-Serayan River Basin

**Fig. 4:**
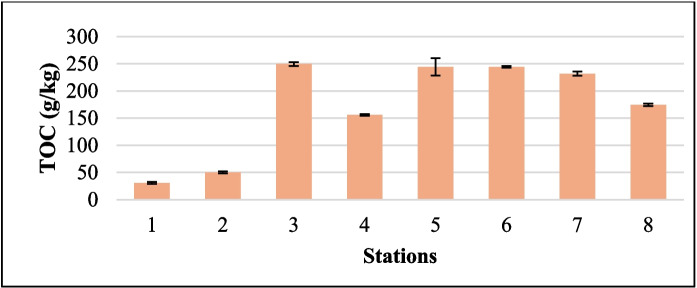
TOC concentrations of the sediments collected from all stations along the Sematan-Serayan River. The unit for TOC is g/kg. The error bars represent the standard deviation

The concentration of TOC varied across the eight sampling stations, ranging from a mean of 30.67 g/kg at S1 to 249.25 g/kg at S3 (Fig. 4). A one-way ANOVA revealed that these differences were statistically significant, F (7, 22) = 672.02, p <.001, indicating substantial variation in TOC levels between stations.

However, Levene's test for homogeneity of variances was also significant (F (7, 22) = 55.24, p <.001), suggesting that the assumption of equal variances was violated. This heterogeneity in variances suggests underlying differences in environmental conditions or sources of organic input across the stations. The TOC was moderately positively correlated with several metals, including As (r = 0.576, p = 0.135), Ni (r = 0.415, p = 0.307), Cr (r = 0.439, p = 0.277), and Cu (r = 0.417, p = 0.304). Although these correlations were not statistically significant (p > 0.05), they suggest a potential influence of organic matter on the distribution and accumulation of these metals. This trend is consistent with the findings by Nenova et al. (2023) and Zhang et al. (2022), who reported that a relatively high TOC content can enhance the retention of heavy metals such as Pb, Cd, and As through the formation of organometallic complexes, which reduce metal solubility and mobility. Furthermore, TOC was strongly correlated with As and Cd (r = 0.111, p = 0.793), metals often associated with organic matter due to their affinity for sorption onto humic substances and precipitation under anoxic conditions typical of mangrove and blackwater environments (Seleghim & Horikawa, 2020).

S1 and S2, located at the saltwater river mouth areas, are likely influenced by stronger hydrodynamic flow and tidal flushing, which reduce organic matter accumulation, including organic carbon accumulation, in the sediment (Che et al., 2023). The elevated TOC at S3 and S4 corresponds with their proximity to mangrove habitats, which are significant carbon sinks because of their dense vegetation, accumulation of organic detritus, and anoxic conditions (Adame et al., 2024; Chynel et al., 2022). S6, S7, and S8, which are influenced by blackwater conditions, also exhibited high TOC values (Ukotije-Ikwut et al., 2023). Humic substances in blackwater rivers can increase TOC levels in sediments. Additionally, the limited water exchange and slow flow in these areas further facilitate organic matter retention (Repasch et al., 2022).

### Surface sediment particle sizes

**Fig. 5:**
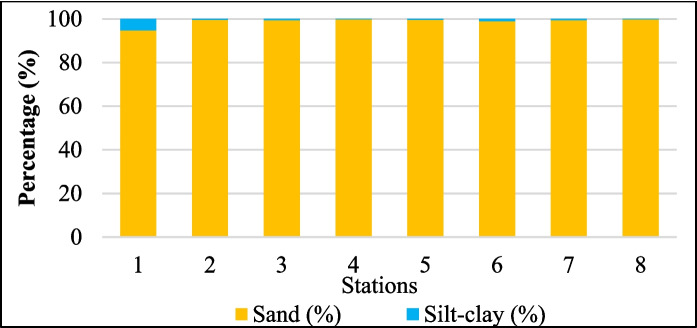
Percentages of particle sizes in sediment samples from all the stations along the Sematan-Serayan River

The sediment composition across the eight stations was dominated by sand, with a mean sand content of 98.91 ± 1.71% and a mean silt–clay content of 1.09 ± 1.71% (Fig. 5). The highest sand content was recorded at S4 (99.80%), whereas the lowest content was recorded at S1 (94.73%), which also exhibited the highest proportion of silt–clay (5.27%). These findings align with the theory that coarser sediments, such as sand, possess lower surface areas and adsorption capacities, limiting their ability to accumulate heavy metals (Table 2) (Duong et al., 2022). However, S1, with the highest silt–clay content, showed elevated levels of metals, such as Fe, Mn, Al, Co, Ni, and Cr. This pattern supports the theory that finer particles provide greater reactive surfaces and are more likely to adsorb and accumulate metals through complexation with organic matter or oxides (Shen et al., 2020). S6 had the highest concentrations of Cu, Fe, Pb, Ni, Cr, Al, and As. Despite having the second-highest silt–clay percentage (1.01%), its metal enrichment exceeds that of S1. These findings suggest that factors other than particle size, such as proximity to pollution sources, organic content, redox conditions, and hydrodynamic impacts, may affect sediment heavy metal deposition (Shobier, 2022).

Thus, while sediment particle size is important, variations in metal concentrations may be due to physical and anthropogenic factors. All locations, except S1, contained over 98% sand, indicating well-sorted, coarse sediments in high-energy coastal and river mouth zones where wave and tidal forces prevent finer particles from settling (Rafati et al., 2022). River mouths in the Kelantan, Pahang, and Endau Rivers display sediment textures ranging from high sand content (> 30%) due to active flow, to silt-dominated sediments (average of 67.2% silt) under low-energy conditions (Liu et al., 2021). However, a limitation of our study is that wet sieving was not conducted during the laboratory work because the instrument was faulty and failed to separate the silt and clay fractions. Consequently, some silt and clay particles may have remained trapped within the coarser sieves, potentially underestimating the actual proportion of fine materials. This limitation affects interpretations involving low silt–clay values, and the true extent of fine particle presence may be greater than previously reported, especially at vegetated or low-energy stations.

### Assessment of potential ecological risks

**Fig. 6:**
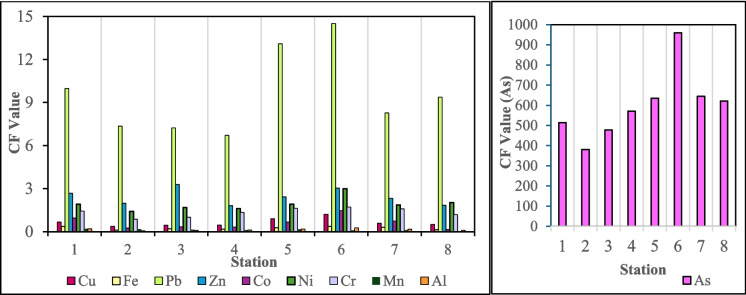
Contamination factor values for the Sematan-Serayan River at all stations

The CF values (Fig. 6) indicate extremely high Pb and As contamination at all stations, with As reaching CF values of 380–960, indicating severe anthropogenic input. S6, influenced by agriculture and sand mining, exhibited the highest As CF (960) and elevated Cd levels (CF = 31.78), while Cd was undetected elsewhere. Zn, Ni, and Cr showed moderate contamination (CF > 1) at S5–S7, whereas Cu, Co, Fe, Mn, and Al remained < 1, reflecting natural background levels. Compared with that in Jakarta Bay, Indonesia (Ikhsani et al., 2025), Zn contamination in this study was moderate but lower (5.21 vs. < 5), Cd was markedly higher (31.78 vs. 2.07), and Pb was far more severe (> 100 vs. 3.17). While the concentrations of Cu and Fe were moderate in Jakarta Bay, both were below 1 here. Ni, which was consistently below 1 in Jakarta Bay, exceeded 1 in this study, indicating anthropogenic enrichment.

**Fig. 7:**
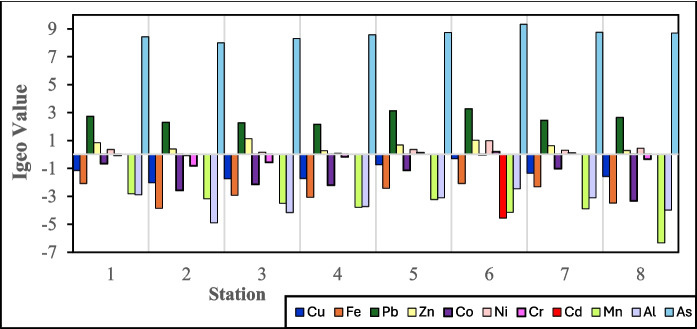
Geoaccumulation index values for the Sematan-Serayan River at all stations

The Igeo values (Fig. 7) mirrored the CF trends, with As consistently showing extreme pollution (7.99–9.32) across all stations. Pb showed moderate to strong contamination (2.16–3.27), whereas Cd was detected only at S6 (–4.56), reflecting a low presence elsewhere. Cu, Fe, Co, Ni, Cr, Mn, and Al exhibited negative Igeo values, indicating background levels. Similar patterns for Ni, Co, Cu, Fe, Mn, and Cr have been reported in the Narew River, Poland, where these metals are unpolluted (class 0) (Skorbiłowicz & Sidoruk, 2024). However, Pb, Zn, and Cd in the Narew River Basin showed only moderate contamination (Igeo = 0.52, 0.35, and 1.05, respectively), attributed to industrial and agricultural inputs. In contrast, the Sematan-Serayan River exhibited considerably higher Pb, extremely elevated As, and Cd occurrence at a single station, suggesting stronger anthropogenic pressure, likely from agricultural runoff, sand mining, and other land-based sources.

The PLI values (Table 3) were > 1 at all stations except S2 and S8. In the Al-Gharraf River, Iraq, the PLI ranged from 0.85 to 1.34, with Cd as the primary pollutant and Ni and Pb as moderate contributors (Al Mayyahi & Al-Shammary, 2022). In contrast, the Sematan-Serayan River contained undetected Cd, except at S6, whose inclusion suppressed PLI values, underscoring how differences in the presence of Cd between regions can substantially influence composite pollution metrics. The RI values (Table 3), which were calculated for six metals with available toxic response factors (Cu, Pb, Zn, Cr, Cd, and As) following Hakanson (1980a, b), placed all the stations in the “very high risk” category (RI > 600). S6 presented the highest risk (10,635.27), driven by extremely elevated As and detectable Cd, followed by S5 (6,421.41), S7 (6,496.24), S8 (6,268.94), and S4 (5,743.61). S1 (5,198.08), S3 (4,813.41), and S2 (3,843.15) also indicated substantial risks from Pb, As, and other metals. In the Bohai Sea, China, 81.25% of sites are classified as very high risk, primarily from Cd, with many exceeding RI = 600 (Liu et al., 2016). Unlike the coastal Bohai Sea, where Cd is dominant, the risk to the Sematan-Serayan River is driven primarily by As and Pb, resulting in generally higher RI values and indicating a more severe ecological threat from heavy metal contamination.

**Table 3:** Pollution load index and potential ecological risk index values for the Sematan-Serayan River at all stations

Station	PLI	Level of pollution	RI	Level of risk
1	1.81	Polluted	5198.08	Very high risk
2	0.94	Unpolluted	3843.15	Very high risk
3	1.20	Polluted	4813.41	Very high risk
4	1.17	Polluted	5743.61	Very high risk
5	1.77	Polluted	6421.41	Very high risk
6	2.85	Polluted	10,635.27	Very high risk
7	1.56	Polluted	6496.24	Very high risk
8	0.93	Unpolluted	6268.94	Very high risk

RI was calculated following Hakanson (1980a, b), using only metals with available toxic response factors (Cu, Pb, Zn, Cr, Cd, and As)

## Conclusion

In conclusion, an alarming concentration of As was recorded at all stations, which led to extremely severe ecological risks. S5 and S6 were involved in sand dredging activities, and the data for most heavy metals were very high at S6. These metals are believed to slowly flow back into the riverbed, thus influencing the biogeochemical behaviour of heavy metals. Positive correlations among several metals, including Cu-Pb, Cu-Ni, Cu-Al, Fe-Al, and Ni-As, suggest a shared origin or common geochemical pathways. The elevated concentrations of Cu, Ni, Pb, and As at certain stations imply anthropogenic influences, particularly from sand dredging activity and agricultural inputs such as fertilisers. The low correlation of Cd and Mn with other metals suggests that they may originate from different sources or are more influenced by redox conditions in the sediment. While this study revealed spatial variation in heavy metal concentrations across sampling stations, TOC levels did not show a strong statistical correlation with heavy metals. Nevertheless, high TOC at some stations may have contributed indirectly to metal retention by promoting reducing conditions and binding with metals. The severity of contamination, which exceeds that reported in other regional and international case studies, points to intense anthropogenic pressure from agriculture, sand mining, and other land-based sources and emphasises the urgent need for targeted pollution control measures.

## Data Availability

No datasets were generated or analysed during the current study.
